# Parameter Dependency of Electrochemical Reduction of CO_2_ in Acetonitrile – A Data Driven Approach

**DOI:** 10.1002/cphc.202400794

**Published:** 2024-12-03

**Authors:** Connor Deacon‐Price, Aleksandra Mijatović, Huub C. J. Hoefsloot, Gadi Rothenberg, Amanda C. Garcia

**Affiliations:** ^1^ Van't Hoff Institute for Molecular Sciences University of Amsterdam Science Park 904 1098 XH Amsterdam The Netherlands

**Keywords:** CO_2_RR, non-aqueous solvent, electrolyte, Multivariate analysis, random forest modelling

## Abstract

The electrochemical CO_2_ reduction reaction (CO_2_RR) is a promising technology for the utilization of captured CO_2_. Though systems using aqueous electrolytes is the state‐of‐the‐art, CO_2_RR in aprotic solvents are a promising alternative that can avoid the parallel hydrogen evolution reaction (HER). While system parameters, such as electrolyte composition, electrode material, and applied potential are known to influence the reaction mechanism, there is a lack of intuitive understanding as to how. We show that by using multivariate data analysis on a large dataset collected from the literature, namely random forest modelling, the most important system parameters can be isolated for each possible product. We find that water content, current density, and applied potential are powerful determinants in the reaction pathway, and therefore in the Faradaic efficiency of CO_2_RR products.

## Graphical abstract

**Branching out**: Using random forest modelling as opposed to conventional linear analysis gives additional insights into the parameters governing the product Faradaic efficiency in aprotic CO_2_RR.

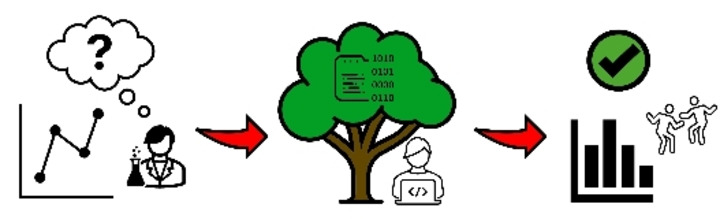

## Introduction

The CO_2_ reduction reaction (CO_2_RR) is an essential step in most processes aimed at converting CO_2_ into a useful product.[^1^, ^2^, ^3^] Considering its scalability and mild reaction conditions, electrochemical CO_2_ reduction is receiving much attention. The most well studied CO_2_RR system is aqueous, using water‐based electrolytes.[^4^, ^5^] Thanks to many rigorous studies and the intense investigation of aqueous electrochemistry, significant progress has been made in understanding the reaction parameters in such systems, particularly in terms of potential dependency,,[^6^, ^7^] pH,[^8^, ^9^, ^10^] and cation effects.[^11^, ^12^, ^13^, ^14^]

However, aqueous CO_2_RR systems have serious inherent drawbacks *because* of the water. The two largest problems are mass transport limitations due to the poor solubility of CO_2_ in water‐based electrolytes,^[15]^ and the concomitant hydrogen evolution reaction (HER).[^16^, ^17^, ^18^] The latter can be partially addressed through electrode design[^19^, ^20^, ^21^, ^22^] and electrolyte control,[^11^, ^13^, ^16^, ^23^] though this limits further tunability and freedom of design within the system.

Alternatively, CO_2_RR can be performed in non‐aqueous electrolytes. The use of aprotic solvents, like acetonitrile (MeCN), for example, offers eight‐fold higher CO_2_ solubility together with the possibility for minimising HER, because of the lack of proton availability.[^24^, ^25^, ^26^, ^27^, ^28^, ^29^] Despite the limited product scope for aprotic CO₂RR, which predominantly yields oxalate, carbon monoxide, and formate (the latter only in the presence of residual water), the parameters governing the various reaction steps are ill‐defined. For instance, product selectivity is highly sensitive to water content[^25^, ^30^, ^31^] and applied potential,[^32^, ^33^, ^34^] yet we still don't know how these parameters influence the reaction pathways.

To address these issues, we gathered a large set of data from the literature on electrochemical CO_2_RR in acetonitrile. We pre‐processed this data and then ran a multivariate analysis, a powerful technique for determining trends in large chemical datasets.[^35^, ^36^] Here we show that random forest modelling *via* ensemble machine learning gives an understanding of the importance of the reaction parameters and their interaction. From this, we can construct a clearer picture of the reaction pathways and product distribution.

## Results and Discussion

We started by collecting a total of 261 CO_2_RR reaction examples from the published literature of the past 50 years, recording nine parameters {salt concentration; cation used; anion used; applied potential; current density; working electrode; solvent; water content; and temperature} and using the Faradaic Efficiencies for oxalate, formate, CO, and hydrogen H_2_ as our figures of merit. This 261×13 data matrix was pre‐processed for robustness and ease of comparison. A detailed description of the data collection and pre‐processing methods, as well as a description of the random forest modelling is included in the methods section. For completeness, we also include a more detailed explanation of the workings of random forest models in the supporting information.

**Figure 1:**
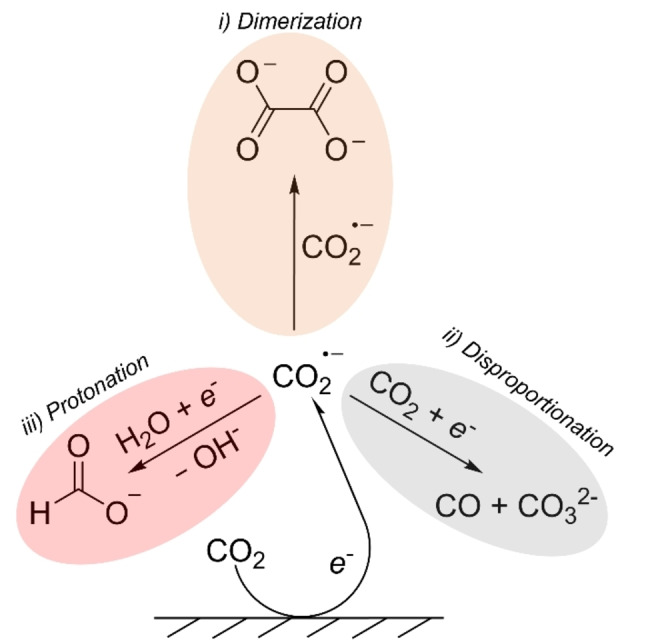
An overview of the three possible CO_2_RR pathways in aprotic solvent: i) dimerization to oxalate, ii) disproportionation to carbon monoxide and carbonate, and iii) protonation to form formate.

**CO_2_ reduction under dry conditions**. In aprotic systems, CO_2_RR follows three major pathways, as described by the Amatore‐Savéant (Figure 1). All of these proceed *via* a common intermediate: the anionic radical CO_2_⋅^−^.[^37^, ^38^] The pathways are therefore in direct competition, and their selectivities are linked; CO_2_⋅^−^ anionic radicals can recombine to yield oxalate (step *(i)* dimerization).[^26^, ^33^, ^38^] This process occurs in the diffuse layer from desorbed CO_2_⋅^−^.^[38]^ Alternatively, the radical anion can react with a CO_2_ molecule and undergo disproportionation, yielding carbon monoxide and carbonate after a second electron transfer (step *(ii)* disproportionation).[^26^, ^37^, ^38^] In practice, residual water is often present in the solvent and therefore a third pathway (step *(iii)* protonation) also takes place, leading to formate.[^24^, ^37^] While we acknowledge that in aqueous systems, CO can be produced via a hydration pathway (CO_2_ + H_2_O + 2e^−^→CO + 2OH^−^), this pathway is not expected to play a significant role in our study. The reasons for this are twofold: first, our studies are specifically designed to minimize water content in acetonitrile to below 1 v/v%, a level at which water‐driven CO_2_RR pathways, including hydration, are highly suppressed.^[39]^ Second, previous studies have shown that in acetonitrile, water tends to form isolated pockets, reducing its interaction with dissolved CO_2_ and favoring HER over CO formation.^[30]^ Consequently, the likelihood of CO production via a hydration mechanism is negligible under the low‐water‐content conditions used in this study.

Only when water is present in quantities, the production of hydrogen through water or proton reduction is favoured.^[24]^ As we recently showed, HER quickly becomes the dominant reaction in acetonitrile electrolytes containing >1 v/v% water.^[40]^

**Figure 2:**
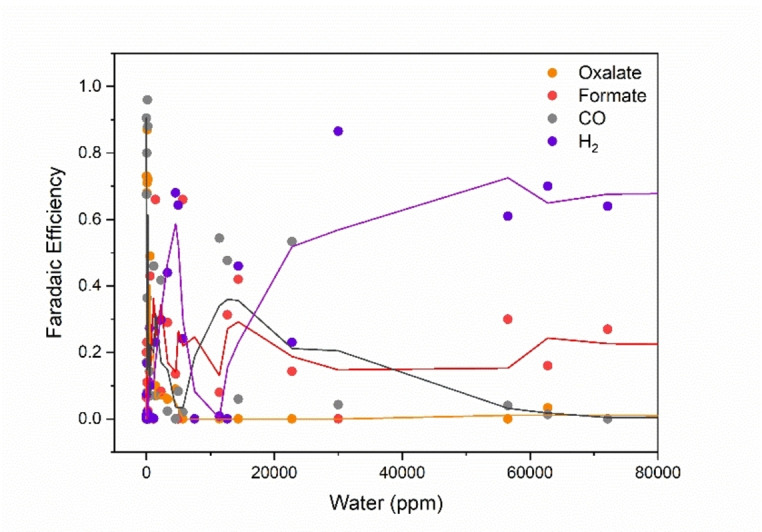
Scatter plot of the simple dependence of FE% on the water content in MeCN, based on the nominal water amounts reported in the data set. Trendlines are calculated as a two‐point moving average.

**Understanding the influence of water**. The water concentration affects both the activity and the product selectivity to oxalate, formate, CO, and H_2_, independent of the cathode. Even considering only the simple dependence of the FE% for the four products on the water content in acetonitrile, we can see some trends (Figure 2). In general, and not surprisingly, higher concentrations of water promote HER, as well as formate production, while dry solvent conditions favour significant oxalate formation.^[28]^ Above 5000 ppm water content, oxalate is no longer produced. The extreme sensitivity to water is understandable, given that protons are very reactive in aprotic media (H^+^ in MeCN is 100 million times more reactive than in water^[41]^). Thus, selective CO_2_RR is only possible in acetonitrile within a very narrow range of water concentrations. As the primary goal of using aprotic solvents is avoiding HER, drying the electrolyte is of utmost importance (more details on properly solvent drying techniques are provided as a technical note in the methods section).

The Amatore‐Savéant mechanism shows that the formate and oxalate pathways are in direct competition. Both involve the CO_2_⋅^−^ radical anion, and both products are generated *via* outer‐sphere electron transfer.[^37^, ^38^] The reason we observe an inverse FE% correlation between oxalate and formate but not with CO is that the final step in the formation of the first two occurs in the diffuse‐reaction layer, while CO forms at the outer Helmholtz plane, an inner‐sphere process.[^29^, ^38^]

This simple correlational analysis of Figure 2 provides some general trends, but the data is still convoluted and non‐linear. Indeed, part of this complexity may be due to hydrodynamics, which can influence the Faradaic Efficiency in electrochemical systems.^[42]^ Our dataset does not take hydrodynamics and cell structure into account. Nevertheless, we felt that a deeper analysis could unravel that part of the complexity that is reflected in the reaction parameters. To this end, we adopted a multivariate approach. We used random forest (RF) modelling. This is an ensemble learning model, building multiple decision trees during training and then merging their outputs to improve accuracy. In RF modelling, datasets are bootstrapped^1^ before many random decision trees are built (hence the name ‘random forest’). The aggregate output is then collected to determine the importance. Performing both dataset bootstrapping and then aggregating the output is called Bagging. In the construction of the RF model, some of the original data is left out, to be used later as test data (the so‐called ‘out of bag’ data, or OOB). Predicting against these actual values determines the OOB Score, which is a measure of the model's predictive power. Similarly, the coefficient of determination for the model data, *R* ^2^, gives an overall metric for the model's robustness.

RF modelling is advantageous in this specific application for several reasons. Ensemble learning methods are typically used on much larger datasets than this one and carry a risk of over‐fitting on small sets. RF models avoid this risk, as they aggregate the results of many decision trees trained on random subsets of the data. Moreover, they can handle non‐linear datasets, accurately predicting the most relevant variables. RF is also compatible with different variable forms, e. g. continuous and categorical. Finally, the model can deal with outliers and high data dimensionality. This is important, because literature datasets are inherently less accurate than ones obtained in a single controlled set of experiments or simulations.

**Figure 3:**
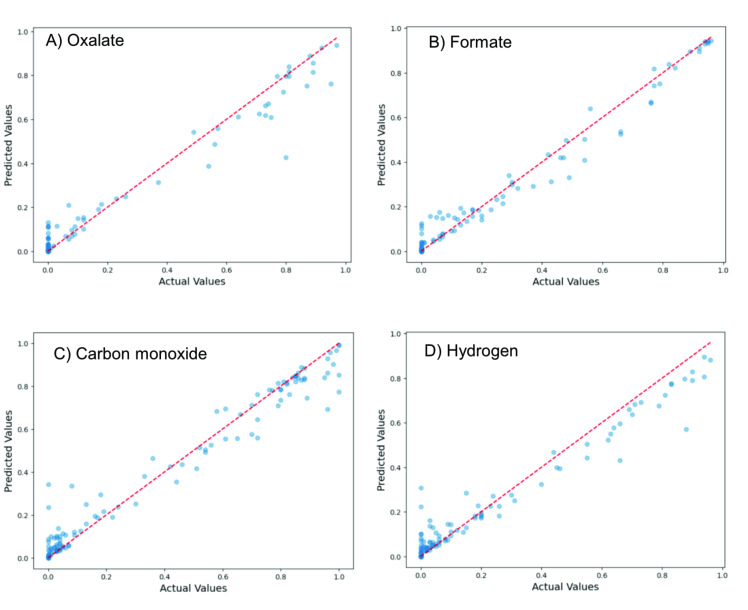
Parity plots showing the random forest model predictions for FE target variables A) Oxalate, B) Formate, C) CO, and D) H_2_.

Figure 3 and Table 1 show the parity plot, *R* ^2^ values and OOB scores for the four products, obtained by the RF models. All four *R* ^2^ values are >0.95, demonstrating a strong correlation within the dataset itself. The OOB scores show that the models are effective at predicting FE, though H_2_ and CO are lower than FA and OA. We can understand this by considering the parity plot prediction at low actual values (bottom‐left side of the plots in Figure 3). At these values, the model consistently predicts too high, suggesting a systemic error. Indeed, one would expect more accurate results for the two liquid products, as these are easily quantified with HPLC. Conversely, unless you have dedicated equipment, measuring gaseous products together with liquid products often results in some gas product loss. Finally, H_2_ is a difficult product to measure with thermal conductivity detectors (TCDs), and in setups tuned to detect CO_2_RR products it often has low responses.

**Table 1:** *R* ^2^ values and out‐of‐bag (OOB) scores calculated for each target variable.

Metric	H_2_	CO	FA	OA
*R* ^2^	0.953	0.960	0.976	0.976
OOB score	0.623	0.710	0.840	0.771

Note that the oxalate RF model predicts lower values than actual compared to formate. This is understandable, because oxalate forms only at very low water concentrations. Most of our data set is outside this range, creating a bias in the model. This is not an issue with formate, which has a higher water tolerance.^[24]^

**Figure 4:**
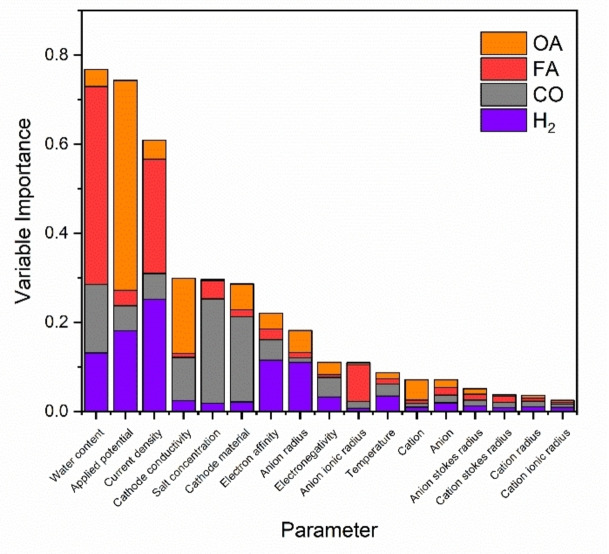
Variable importance (VIP) plot showing the importance values of each parameter in relationship to the predicted for product FE as determined by random forest regression. H_2_=hydrogen; CO=carbon monoxide; FA=formate; OA=oxalate.

**Chemical interpretation of the models**. To elucidate the influence of various reaction parameters, we categorized them into “important” and “less important” groups using a Variable Importance (VIP)^[44]^ plot (Figure 4), where all variables are ranked according to their coefficients.

The VIP plot provides several insights. First, three parameters distinctly stand out: water content, applied potential, and current density. This observation is consistent with our correlational data analysis (Figure 2 and Figure S1). To identify subsequent system dependencies, these three parameters must first be optimized. For instance, any trends in oxalate production relative to the cation will only become apparent when the appropriate potential is applied.

Second, we see that the cathode conductivity and material, and the salt concentration are also relatively important, whereas the anion‐related and cation‐related parameters have little to no influence on the product selectivity in this system.

**Figure 5:**
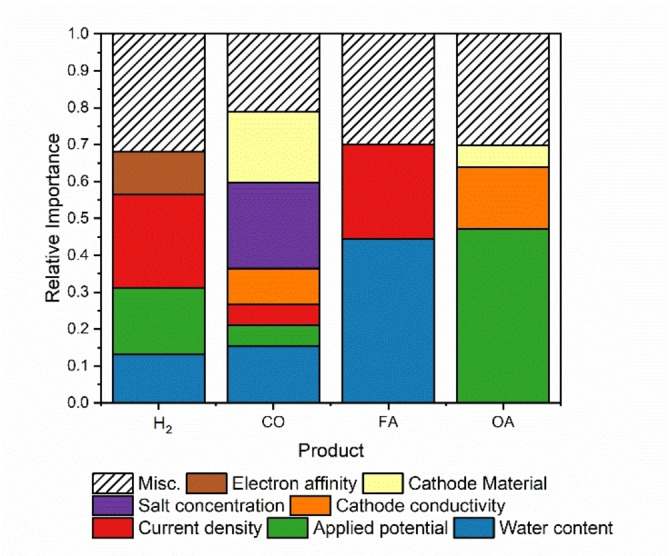
The relative importance values of the key parameter in relationship to the predicted for product FE as determined by random forest regression. Parameters accounting for <5 % are combined as a single parameter, Misc. H_2_=hydrogen; CO=carbon monoxide; FA=formate; OA=oxalate.

We can gain more insight by discarding the less important variables and transposing the column graph (Figure 5). Here, we focus only on the main variables, aggregating all the others as “miscellaneous”. We now see that in the case of oxalate and formate, 65–70 % of the FE can be predicted by just two parameters. Formate is predominantly affected by the water content and current density. This is logical as water is required as a hydrogen source, and higher current densities give higher local CO_2_⋅^−^ concentrations.^[38]^ Given that water is very reactive in aprotic media,^[41]^ a high reaction rate correlates with a high selectivity to formate. Conversely, oxalate is most sensitive to the applied potential and the cathode conductivity. This is interesting, as water content is a less important than what we would initially expect. However, this may be a limitation of the model itself (see above). Conversely, applied potential is a logical parameter, as CO_2_⋅^−^ radicals must be present to promote radical recombination and dimerization for forming oxalate. Cathode conductivity is also strongly correlated in generating oxalate. This may reflect rapid electron transfer, enabling higher local concentrations of CO_2_⋅^−^ radicals, which would be improved when using highly conductive materials.

**Figure 6:**
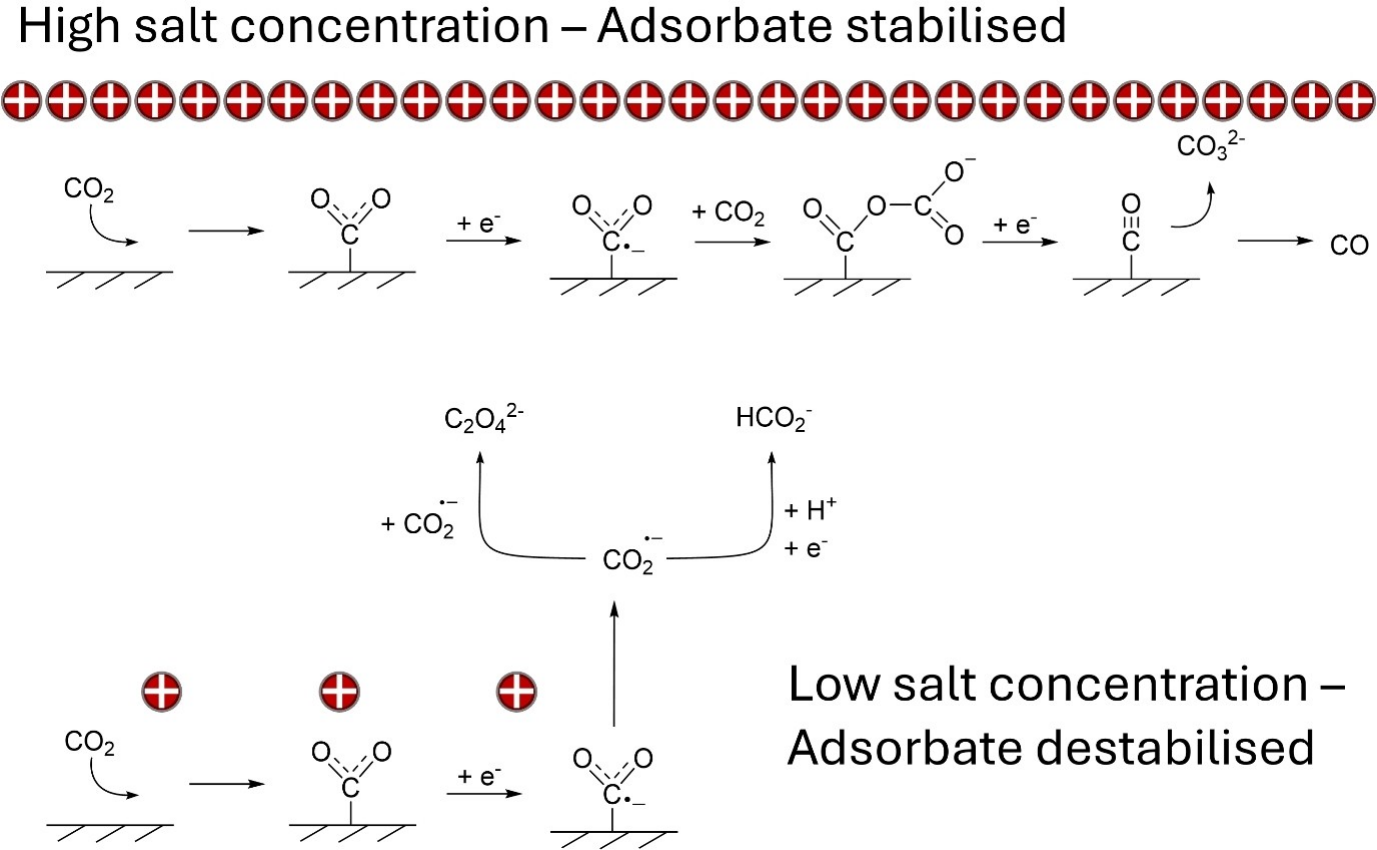
Suggested pathways showing how salt concentration may promote CO production via disproportionation compared to dimerization to oxalate and protonation to formate, respectively.

CO selectivity is also influenced by cathode conductivity. However, it also depends on the salt concentration and the cathode material. 60 % of the FE prediction is described by these three parameters. We suggest that salt concentration is predictive as the disproportionation reaction to generate it likely occurs in the outer Helmholtz plane.^[29]^ This is in contrast to the formation of oxalate and formate, which occur in the diffuse layer.[^29^, ^38^] Since surface adsorbed CO_2_RR requires stabilisation of the reaction intermediates *via* the cation,[^14^, ^45^, ^46^] a low availability of cations would limit this effect. This in turn would promote desorption towards diffuse layer products, such as formate and oxalate (Figure 6).

The fact that the disproportionation pathway requires adsorbed intermediates explains the importance of the cathode material. Different cathode materials have differing capacities at which they can stabilise intermediates at their surfaces.^[47]^ Pb electrodes are noted for promoting oxalate formation due to their inert properties and high HER overpotential, which prevent interference with other CO_2_RR pathways and side reactions.[^32^, ^48^] In contrast, strongly adsorbing materials like Pt and Au promote CO formation by blocking active sites.[^31^, ^38^, ^49^] However, some studies indicate Pt electrodes may also favour oxalate formation, but primarily at very high overpotentials.[^24^, ^50^, ^51^] Finally, the relative importance of the water content may be due to the contribution of aqueous pathways towards CO. In aqueous systems, CO generation requires just two molecules of water to strip an oxygen atom off the CO_2_ adsorbate.^[52]^ The scaling relations involved in generating more complex and protonated species from the CO_2_RR become limiting. Additionally, in aqueous systems with tetraalkylammonium electrolyte, which are the most used supporting electrolyte in aprotic media, CO was the main product.^[53]^ Since protonated C_2+_ products are only favourable in aprotic media when using high surface area cathodes or high pressures,^[29]^ the CO_2_RR would likely stop at CO production. For these reasons, if aqueous CO_2_RR does take place, CO is the most likely product.

**Figure 7:**
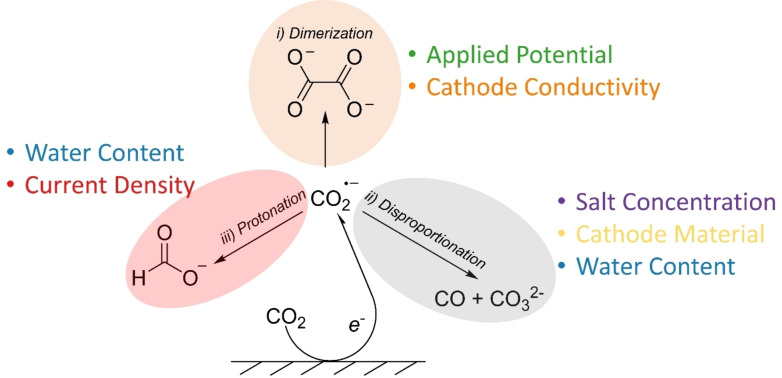
The key parameters in determining FE for each product as predicted by random forest modelling.

Similarly, H_2_ FE is also described by the water content, the applied potential, the current density, and the cathode electron affinity. However, as the model prediction quality of the H_2_ FE is moderate, one should be careful with interpretations here. The sensitivity to water content is logical, as it is required to produce H_2_. Similarly, more negative potentials and higher current densities are indicative of HER.[^28^, ^40^] Figure 7 summarises the most important features in predicting the FE of the CO_2_RR products in acetonitrile‐based electrolytic solution.

## Conclusions

We performed correlational and multivariate data analysis on a pre‐processed dataset gathered from the literature regarding CO_2_RR in acetonitrile electrolytes. We found trends in the applied potential and water content in the correlational data analysis, but at this level of dimensionality any other trends are difficult to identify. This we solved by running random forest modelling to understand the importance of each tuneable parameter as a function of the product FE. With this model, we identified three major factors in determining aprotic CO_2_RR: the water content, the applied potential, and the current density. Formate and oxalate are mostly described by just two parameters, whilst H_2_ and CO depend on several parameters. Our results show the value of using multivariate models for extracting trends from electrochemical data and give insight as to the parameters governing the different pathways possible in CO_2_RR. We hope that these results will encourage more scientists to use such methods in their research.

## Experimental Section

**Data collection and pre‐processing**. Data was collected by searching the Chemical Abstracts service SciFinder for peer‐reviewed journal papers in English and German containing the terms “CO_2_ reduction” and “acetonitrile”. The publications ranged from 1973 to 2023. These papers were sifted manually for data, recording nine parameters {salt concentration; cation used; anion used; applied potential; current density; working electrode; solvent; water content; and temperature}.[^24^, ^28^, ^32^, ^34^, ^48^, ^49^, ^51^, ^54^, ^55^, ^56^, ^57^, ^58^, ^59^, ^60^, ^61^, ^62^, ^63^, ^64^, ^65^, ^66^, ^67^, ^68^, ^69^, ^70^, ^71^] The Faradaic efficiencies (FE%, the electrochemical equivalent of product selectivity) were set as the *figures of merit* (also known as the *target variables*) for four products {oxalate (OA); formate (FA); carbon monoxide (CO), and hydrogen (H_2_)}. Initially, we also included methane but disregarded it owing to the lack of data on its formation. This resulted in a 261×13 data matrix (9 variables and 4 figures of merit).

The ferrocene/ferrocenium couple, Fc/Fc^+^, is the recommended IUPAC standard couple. However, as very few Fc/Fc^+^ were reported, we converted all the reported applied potential values to Ag/Ag^+^ according to the reference potentials. We then translated all the categorical data into quantitative values using descriptors.^[72]^ Cation and anion species were described using their covalent, ionic, and Stokes radii (Å). Similarly, working electrodes were described in terms of electronegativity, electron affinity (eV), and electrical conductivity (mS/m). For working electrodes comprising multiple elements we used a mean based on the atomic ratios. A detailed list of the literature and methods used for determining these values is included in the SI (Table S1–S3). Where no parameters were reported, these values were left as null. Finally, we removed those experiments where the total sum of the FE% was <75 %. These inputs were deemed as unreliable as such low values would not give a robust model.

**Random forest modelling**. The random forest model was created using Python 3, with NumPy, Pandas, and PyPlot and Random Forest Regressor (Scikit‐Learn) imported packages. The pre‐processed dataset was imported as a .csv file and interpreted as a data frame. We selected the target variable as one of the FE% values to be read as the y axis and defined the x variables as all the descriptors in the data frame. Null data points were set to the mean value of the parameter. We then split the data frame into a training set (80 %) and a test set (20 %). The data was then standardised using the Standard Scaler function, ensuring that all variables contribute equally to the model training. The target variable was then flattened, after which we ran the random forest regressor. The random state was set to 42,^[73]^ and the Out‐Of‐Bag (OOB) score enabled, printing the resulting importance values and model OOB score. The parity plot was also printed, showing predicted vs actual values after training, as well as the *R* ^2^ value. No hyperparameters were modified for this study. A full description of random forest modelling is included in the SI.

**Figure 8:**
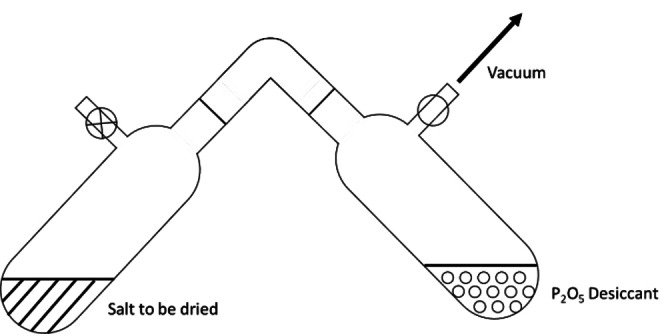
Drying a salt over a P_2_O_5_ desiccant under constant vacuum on a Schlenk line.

**A technical note on the drying of aprotic electrolytes**. Most tetraalkylammonium salts are hygroscopic, adding unwanted water to the electrolyte under preparation. Desiccating these salts is a good strategy to limit this. Vacuum ovens (with temperatures below degradation temperatures of the salt in question) are effective for this purpose, or desiccation *via* a Schlenk line under vacuum (Figure 8). For optimal drying, glassware should be flamed under vacuum and allowed to cool under dry and inert gas before introducing the salt. Desiccant choice depends on the required level of drying and the hygroscopicity of the salt. However, we recommend using P_2_O_5_ for optimal drying. Under vacuum, absorbed moisture in the salt will gradually evaporate, entering the gas phase. Contact with P_2_O_5_ will trap the water chemically as phosphoric acid (eq 1). This is a slow procedure, and the longer it is allowed to remain under vacuum the drier the final sample will be. We recommend at least 24 h. **CAUTION**! P_2_O_5_ is extremely reactive. Its reaction with water is exothermic – handle with care.
(1)

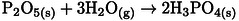




Using zeolite molecular sieves is another effective method for drying electrolytes. These materials have well defined pore sizes, allowing the separation of chemicals by size on a molecular level. The kinetic diameter of MeCN is ~6.5 Å,^[74]^ whilst that of water is ~2.8 Å.^[75]^ This means that a 3 Å pore size molecular sieve will selectively filter out the water. Such 3 Å molecular sieves outperform other methods of drying in almost all cases.^[76]^ A circumstance where molecular sieves are not appropriate is when other non‐impurity species are present in solution which are smaller than the pore size, as they will also be filtered. Another downside of using molecular sieves use is that if aiming for very pure samples, trace impurities may be conferred by the molecular sieves to the electrolyte, though suitable cleaning will mitigate this. We recommend leaving these electrolytes to dry under an inert gas for 24 h before use.^[76]^ Once removed from a Schlenk line and exposed to the atmosphere, MeCN electrolytes will immediately begin to absorb water. To avoid this, one should use flamed or suitably baked glassware with a constant pressure of dry gas flowing through them.

## Conflict of Interests

The authors declare no conflict of interest.

1

## Supporting information

As a service to our authors and readers, this journal provides supporting information supplied by the authors. Such materials are peer reviewed and may be re‐organized for online delivery, but are not copy‐edited or typeset. Technical support issues arising from supporting information (other than missing files) should be addressed to the authors.

Supporting Information

## Data Availability

The data that support the findings of this study are available from the corresponding author upon reasonable request.

## Footnotes

n1 Re‐sampling of the dataset in different ways, or *bootstrapping*, is like cross‐validation. The idea is that a good dataset should be representative of the total population. Entries from the dataset are selected at random and divided in subsets. Some entries may appear in several subsets, while others may not appear at all. Then, some of the subsets are used for building a model. The remaining results are then predicted using this model. A high average *q* ^2^ value indicates the model's robustness.^[43]^
