# Supplementary material for: Parameter Dependency of Electrochemical Reduction of CO2 in Acetonitrile – A Data Driven Approach
Source: Chemphyschem. 2024 Dec 3;26(4):e202400794. doi: 10.1002/cphc.202400794 (PMC11832059; doi:10.1002/cphc.202400794)
Supplement: Supplementary file 1 — Supporting Information [file CPHC-26-e202400794-s001.pdf]

ChemPhysChem

Supporting Information

Parameter Dependency of Electrochemical Reduction of CO_2 in Acetonitrile – A Data Driven Approach

Connor Deacon-Price, Aleksandra Mijatović, Huub C. J. Hoefsloot, Gadi Rothenberg, and Amanda C. Garcia*

The Parameter Dependency of Electrochemical Reduction of CO_2 in Acetonitrile – A Data Driven Approach

Supplementary Information

Connor Deacon-Price, Aleksandra Mijatović, Huub C.J. Hoefsloot, Gadi Rothenberg Amanda C. Garcia*

Van 't Hoff Institute for Molecular Sciences, University of Amsterdam, Science Park 904, 1098 XH, Amsterdam, The Netherlands

(*) Corresponding author: a.c.garcia@uva.nl

Random forest modelling is a powerful tool in multivariate data analysis. Random forests are composed of decision trees (Scheme S1). Decision trees are very useful ways of sectioning data for string predictive power. Decision trees are easy to build and interpret but suffer from poor inaccuracy when working with non-fixed datasets. They can predict target variables within a dataset that was used to create them with high accuracy but are not flexible when new datasets are applied. Random forests remedy this issue whilst maintaining high predictive power.

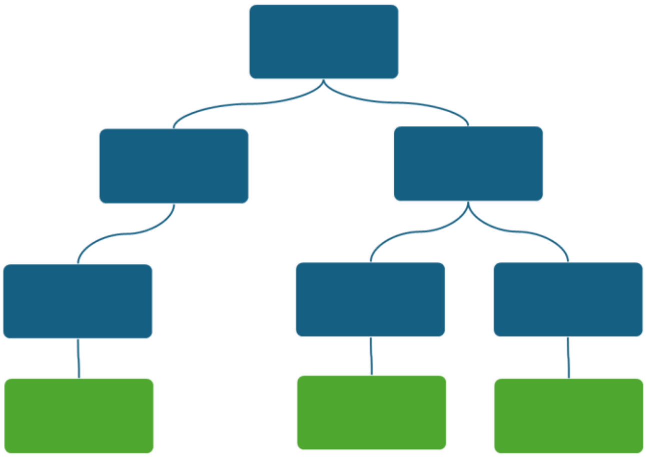

Scheme S1. An example of a decision tree. Blue boxes indicate parameter variables, green boxes indicate target variables.

To create a random forest, the data set must first be boot-strapped. Data samples are selected at random, and samples can be selected more than once. These random samples are then added to a boot-strapped dataset (Scheme S2).

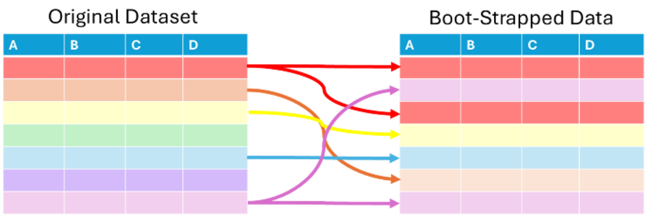

Scheme S2. An example of boot-strapped data and how it is produced at random from a dataset.

A decision tree is then created using the input of this boot-strapped dataset, using random variables at each step of the tree. This includes both x (parameter) and y (target) variables. This is then repeated numerous times, creating many different decision trees with new bootstrapped data with random variables at each step. This collection of decision trees is the random forest (Scheme S3).

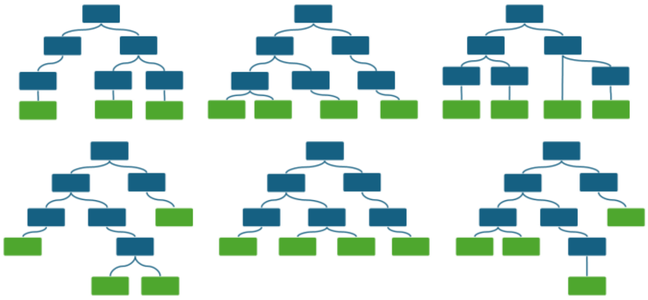

Scheme S3. An example of a random forest constructed from multiple randomly generated decision trees from boot-strapped data.

The random forest then uses runs new data through this collection of decision trees to determine the target variable value as an aggregate. Process of bootstrapping and taking the aggregate result across the random forest is called Bagging. To test the predictive accuracy of the random forest model, data that was in the original dataset but was not recorded for the creation of the decision trees which compose the random forest, is then ran through the random forest. This is known as the Out-Of-Bag (OOB) data (Scheme S4).

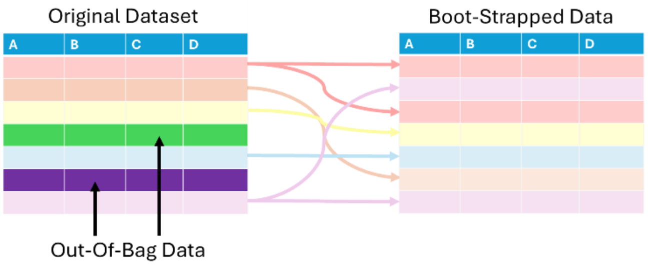

Scheme S4. An example of Out-Of-Bag (OOB) data and how it is selected from an original dataset in respect to the boot-strapped data

The random forest model will make a prediction of the OOB target variable based on the prior bagged data. Based on the aggregate of the data a value is assigned and then compared to the real value. We can use the proportion of correctly assigned values to

construct a measure of model accuracy, known as the OOB Score. OOB Scores which approach a value of 1 indicate very strong models.

This process is repeated many times, constantly bootstrapping and bagging data, and then testing the accuracy of the created random forests. This is done until the random forest is optimised and the highest OOB Score is reached.

Table S1. An overview of the literature used to obtain relevant parameter values regarding the cation identity.

Cation Identity	Cation Radius	Cation Ionic Radii	Cation Stokes Radius
TMA	3.20[1]	2.80[2]	1.82[3]
TEA	4.5[4]	3.37[2]	2.50[3]
TBA	3.87[5]	4.94[5]	3.84[5]
Li	1.45[6]	0.69[2]	0.71[7]
EMIM			
123TMI			
BMIM	3.3[8]	1.6[9]	1.27[8]
1M3PentI			

Table S2. An overview of the literature used to obtain relevant parameter values regarding the anion identity.

Anion Identity	Anion Radius	Anion Ionic Radii	Anion Stokes Radius
ClO_4	3.50[10]	2.25[11]	1.37[12]
PF_6	2.54[13]	2.54[14]	2.28[15]
TFMS	2.7[16]		
Cl	3.00[10]	1.81[2]	1.33[12]
TFAc	2.38[17]		3.48[18]
C_2N_2			
SCN	3.50[10]	2.09[11]	1.4[12]
NO_3	3.00[10]	2[11]	1.29[12]
H_2PO_4	4.00[10]	2.13[11]	1.68[12]
TFB	2.01[19]	4.02[19]	1.68[19]

Table S3. An overview of the literature used to obtain relevant parameter values regarding the working electrode identity. Notes state if the value was taken for a component of the electrode material, or as an average as according to the atomic ratio.

**Electrodes**	**Electronegativity**	**Notes**	**Electron affinity (eV)**	**Notes**	**Conductivity (MS/m)**	Notes

Pb	**2.33**		**0.364**		**4.8**	
MoO2@Pb	**1.89**	MnO_2	**1.5**	MnO_2	**4.8**	Pb
SS	**1.804**	Average	**0.3633**	Average	**1.804**	Average
Sn	**1.96**		**1.112**		**9.1**	
GO/MWCNT-CP			**4.2**[20]		**100**[21]	
Carbon Paper	**2.55**	Carbon	**1.595**	Carbon	**0.1**	Carbon
Au	**2.54**		**2.309**		**45**	
Ag	**1.93**		**1.302**		**62**	
Pt	**2.28**		**2.128**		**9.4**	
Cu	**1.9**		**1.227**		**59**	
Fe4.5Ni4.5S8	**2.217352941**	Average	**1.324323529**	Average	**6.352941176**	Average
Fe4.5Ni4.5S9	**2.2375**	Average	**1.36575**	Average	**6**	Average
Fe4.5Ni4.5S10	**2.255526316**	Average	**1.402815789**	Average	**5.684210526**	Average
CuNC	**1.9**	Cu	**1.227**	Cu	**59**	Cu
Fe4.5Ni4.5S7Se	**2.215588235**	Average	**1.321382353**	Average	**6.352941176**	Average
Fe4.5Ni4.5S6Se2	**2.213823529**	Average	**1.318441176**	Average	**6.352941176**	Average
Fe4.5Ni4.5S5Se3	**2.212058824**	Average	**1.3155**	Average	**6.352941176**	Average
Fe4.5Ni4.5S4Se4	**2.210294118**	Average	**1.312558824**	Average	**6.352941176**	Average
Fe4.5Ni4.5S3Se5	**2.208529412**	Average	**1.309617647**	Average	**6.352941176**	Average
Ga	**1.81**		**0.3**		**7.1**	
Ga/C-CP	**1.81**	Ga	**0.3**	Ga	**7.1**	Ga
Co3O4 nanofiber						
Fe_MOF-525@FTO						
CuSn	**1.927272727**	Average	**1.174727273**	Average	**36.31818182**	Average
Ti	**1.54**		**0.079**		**2.5**	
Nb	**1.6**		**0.892**		**6.7**	
Cr	**1.66**		**0.666**		**7.9**	
Mo	**2.16**		**0.745**		**20**	
Fe	**1.83**		**0.163**		**10**	
Pd	**2.2**		**0.557**		**10**	
Ni	**1.91**		**1.16**		**14**	
Zn	**1.65**		**0**		**17**	
Cd	**1.69**		**0**		**14**	
Hg	**2**		**0**		**1**	
In	**1.78**		**0.3**		**12**	
Tl	**1.62**		**0.199**		**6.7**	
Fe4.5Ni4.5S11	**2.27175**	Average	**1.436175**	Average	**5.4**	Average
Fe4.5Ni4.5S12	**2.286428571**	Average	**1.466357143**	Average	**5.142857143**	Average

Fe4.5Ni4.5S13	**2.299772727**	Average	**1.493795455**	Average	**4.909090909**	Average
Fe4.5Ni4.5S14	**2.311956522**	Average	**1.518847826**	Average	**4.695652174**	Average
Fe4.5Ni4.5S15	**2.323125**	Average	**1.5418125**	Average	**4.5**	Average
Fe4.5Ni4.5S16	**2.3334**	Average	**1.56294**	Average	**4.32**	Average
Fe4.5Ni4.5S17	**2.342884615**	Average	**1.582442308**	Average	**4.153846154**	Average
Fe4.5Ni4.5S18	**2.351666667**	Average	**1.6005**	Average	**4**	Average
Fe4.5Ni4.5S19	**2.359821429**	Average	**1.617267857**	Average	**3.857142857**	Average
Fe4.5Ni4.5S4Se5	**2.229166667**	Average	**1.351861111**	Average	**6**	Average
Fe4.5Ni4.5S4Se6	**2.246052632**	Average	**1.387026316**	Average	**5.684210526**	Average
Fe4.5Ni4.5S4Se7	**2.26125**	Average	**1.418675**	Average	**5.4**	Average
Fe4.5Ni4.5S4Se8	**2.275**	Average	**1.447309524**	Average	**5.142857143**	Average
Fe4.5Ni4.5S4Se9	**2.2875**	Average	**1.473340909**	Average	**4.909090909**	Average

The Random Forest Model Code:

```
import numpy as np
import pandas as pd
from matplotlib import pyplot as plt
import sklearn
from sklearn.preprocessing import LabelEncoder

# importing or loading the dataset
df = pd.read_csv("C:/Users/cdeacon/Downloads/LitReviewProcessed Data Excl low FE.csv")
y = df.iloc[:, -1:].values
df["Voltage/V (vs Ag/Ag+)"] = pd.to_numeric(df["Voltage/V (vs Ag/Ag+)"])
for col in df.columns:
    if col in ["Cation", "Anion", "Working electrode"]:
        continue
    print(col)
    df.loc[df[col] == "x", col] = np.mean(df.loc[df[col] != "x", col])
    df.loc[df[col].isnull() == True, col] = np.mean(df[col])
def encode_df(dataframe):
    le = LabelEncoder()
```

```
    for column in ["Cation", "Anion", "Working electrode"]:
        dataframe[column] = le.fit_transform(dataframe[column])
    return dataframe
#encode the dataframe
df = encode_df(df)
df.head()
X = df.iloc[:, 0:-5].values
X_cols = df.iloc[:, 0:-5].columns
X.shape

# Splitting the X and Y into the
# Training set and Testing set
from sklearn.model_selection import train_test_split

X_train, X_test, y_train, y_test = train_test_split(X, y, test_size = 0.2, random_state = 0)

# performing preprocessing part
from sklearn.preprocessing import StandardScaler
sc = StandardScaler()

X_train = sc.fit_transform(X_train)
X_test = sc.transform(X_test)

from sklearn.ensemble import RandomForestRegressor
import pandas as pd
from sklearn.metrics import r2_score

# Assuming X_train is your input features dataframe and y_train is your target variable
y_train = y_train.flatten()

# Initialize the RandomForestRegressor with oob_score set to True
model = RandomForestRegressor(oob_score=True, random_state=42)

# Fit the model on the training data
model.fit(X_train, y_train)
```

```
54.
55. # Get feature importances
56. importances = model.feature_importances_
57. feature_importance_df = pd.DataFrame({'Feature': X_cols, 'Importance': importances})
58. feature_importance_df.sort_values(by='Importance', ascending=False, inplace=True)
59. print(feature_importance_df)
60.
61. # Compute and print the OOB error
62. oob_score = model.oob_score_
63. oob_error = 1 - oob_score
64. print(f"OOB Score: {oob_score}")
65. print(f"OOB Error: {oob_error}")
66.
67. # Calculate R^2 score
68. r2 = r2_score(y_train, y_pred)
69. print(f"Coefficient of Determination (R^2): {r2}")
70.
71. import matplotlib.pyplot as plt
72.
73. # Assuming y_pred are the predicted values from the model and Y are the true values
74. y_pred = model.predict(X_train)
75.
76. plt.figure(figsize=(8, 6))
77. plt.scatter(y_train, y_pred, alpha=0.5)
78. plt.plot([min(y_train), max(y_train)], [min(y_train), max(y_train)], color='red', linestyle='--')
79. plt.xlabel('Actual Values')
80. plt.ylabel('Predicted Values')
81. plt.title('Predicted vs. Actual Values')
82. plt.show()
```

The Random Forest Model Code - Details:

Lines 1 – 5 import the relevant packages required for the data processing and subsequent modelling. Line 8 imports the dataset, a .csv file, and parses it as a dataframe. In line 9 the y value (the target value) is defined, in this example as the last column of the dataframe. Line 10 ensures that the negative values in the applied

voltage column are stored as numeric values. Lines 11 - 16 selects columns excluding non-integer values and sets null values equal to the mean of the respective column. Lines 17 - 21 label categorical values as unique numbers, assigning each categorical a number, which is verified with line 23 and 24. Lines 25 and 26 define the x values (parameters) as all columns before the 5th column, and the resulting X area is checked with line 27. Data training packages are imported (line 31) and line 33 splits the X matrix and y variable into training (80%) and testing (20%) sets. Reproducibility is ensured with random_state = 0. This code allows for training the model on X_train and y_train, and evaluating its performance on X_test and y_test. The scaling package is imported and defined (lines 36 and 37), before applying this to both the train and test data arrays (lines 39 and 40). This is standardises the data by calculating the mean and standard deviation within the dataset. The random forest regressor package is imported along with the coefficient of determination package (lines 42 – 44). The training data for y is then flattened (line 47). This converts the data from a multi-dimensional array to a one-dimensional array. This allows for its use in machine learning algorithms that expect a one-dimensional array for target variables. Line 50 initialises the random forest regressor with OOB scoring enabled. The random state number is arbitrary, but must be fixed for reproducibility purposes. Line 53 then trains the model on the data array, and the resulting importance values are organised and printed (lines 56 - 59). The OOB score and OOB error are printed (line 62 – 65) alongside the coefficient of determination (lines 68 and 69). The predicted values for y are then defined (line 74) and then plotted against the actual values (lines 76 – 82). This regression scatter plot provides a visualisation of the model strength.

Bivariate Analysis - Potential influence

There is a distinct difference in product distribution between dry and wet MeCN conditions. In order to analyse other variables, it is valuable to separate the data into dry and wet categories. We define dry conditions as MeCN electrolyte with a concentration of water below or equal to 10,000 ppm, in order to strike a balance between data availability and dryness of solvent. In addition, this threshold value is where the water concentrations confer significant differences in the FE% values (Figure 2). The trends regarding the potential influence are presented in Figure S1.

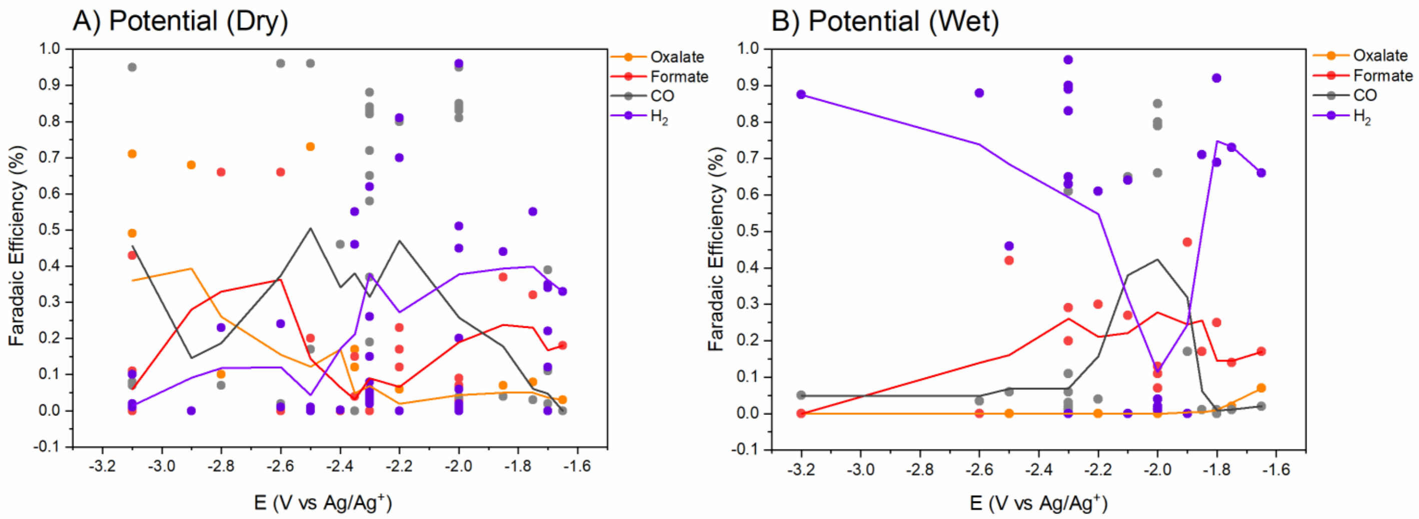

Figure S1. The dependence of FE% on the potential in A) dry (≤10,000 ppm) MeCN, and B) wet (<10,000 ppm) MeCN. Trendlines are calculated as a 2 moving point average.

Oxalate is only detected in considerable amounts in only dry condition and significantly negative potentials (Figure S1A). A wide range of oxalate FE% are detected at these potentials, which is likely a feature of the extremely narrow water content window in which oxalate can be produced. However, considering the Amatore-Savéant mechanism, it is logical that very negative potentials will favour oxalate production. If the local environment is concentrated with $CO_2^{\bullet-}$ radicals, which will increase dramatically (until mass transport limitations are reached) with the applied potential, the likelihood of radical recombination will rise. This is why a strong relationship with this variable is visible. It is also clear that formate and H_2 yields are coupled, following the same pattern until -2.4 V, though formate is half the magnitude. These two products are the only two possible protonated products. More negative than -2.4 V and formate begins to be far more promoted than H_2. We propose that this is likely due to the availability of $CO_2^{\bullet-}$ radicals. At more positive potentials, $CO_2^{\bullet-}$ radicals are limited, meaning that most interfacial water will undergo HER. However, as potentials become more negative, $CO_2^{\bullet-}$ radical concentrations increase, meaning water is more likely to react with $CO_2^{\bullet-}$ rather than itself at the interface. Given that water content is limited in these conditions, $CO_2^{\bullet-}$ radicals may be present at higher local concentrations,

allowing CO2RR to outperform HER. In general, as more negative potentials are applied and CO2RR pathways become feasible, the HER is suppressed.

On the other hand, in dry but more positive potentials other products are favoured. H_2 production occurs at more positive potentials before giving way to CO and formate production. In contrast, despite an anomalously low H_2 production at -2.0 V, HER dominates when electrolytes are wet (Figure S1B). This hints that HER production is decoupled from applied potentials, so long as sufficient potential to drive HER is achieved, if electrolytes are wet. This indicates a turning point where if water content becomes so very potential effects will begin to take hold. Formate production is consistently moderate at around 25%, until extremely negative potentials are reached and HER approaches 100% FE%. CO production peaks at approximately -2.0 V, which is slightly more negative than where CO_2RR typically begins in aprotic solvents.[22–24] Despite very negative potentials where HER dominates in wet conditions, CO production is unique in that its FE% is relatively stable. Once CO_2RR becomes viable it remains a major product, decoupled from the applied potential. This being said, we also observe a large degree of variance in the dataset. This is indicative of another variable dictating CO FE%.

In summary, correlational analysis shows that higher water contents unequivocally promotes the formation of H_2 from the concomitant HER, while oxalate formation reaches high yields only under exceptionally dry conditions, as the present water would otherwise intercept $CO_2^{\bullet-}$ radicals required for it. As another possible product from the aprotic pathway, CO was shown to be less sensitive to the water content compared to the oxalate and also unlikely to be produced in notable amounts, as protic pathways dominate in its place. Similarly to HER, formate also appears to be favoured with increasing concentration of water, but FE%s begin decrease at higher values as H_2 production is favoured instead.
from the exceptionally dry conditions, oxalate also requires very negative potentials due to the $CO_2^{\bullet-}$ radicals requiring rather negative potentials for their generation, as opposed to H_2 which is favoured at more positive values of potential in dry MeCN. The applied potential does not seem to affect H_2 quantities in wet conditions, as it reaches high FE% regardless of the potential value. Formate and CO demonstrate similar overall trends in both dry and wet MeCN, with lower yields in wet solvent owing to the notable promotion of HER.

References

[1] D. H. Aue, H. M. Webb, M. T. Bowers, *J. Am. Chem. Soc.* **1976**, *98*, 318–329.

[2] S. Kubota, S. Ozaki, J. Onishi, K. Kano, O. Shirai, *Anal. Sci.* **2009**, *25*, 189–194.

[3] S. Ding, F. Sachs, *J. Membr. Biol.* **1999**, *172*, 215–223.

[4] E. R. Nightingale, *J. Phys. Chem.* **1959**, *63*, 1381–1387.

[5] L. Sun, X. Liang, N. von Solms, G. M. Kontogeorgis, *Fluid Phase Equilib.* **2019**, *486*, 37–47.

[6] S. Manna, D. Roy, S. Das, B. Pathak, *Mater. Adv.* **2022**, *3*, 7833–7845.

[7] K. Hayamizu, Y. Chiba, T. Haishi, *RSC Adv.* **2021**, *11*, 20252–20257.

[8] N. R. Pitawela, S. K. Shaw, *ACS Meas. Sci. Au* **2021**, *1*, 117–130.

[9] N. Hatano, T. Takekiyo, H. Abe, Y. Yoshimura, R. W. Berg, *Int. J. Spectrosc.* **2011**, *2011*, 648245.

[10] J. G. Speight, *Lange's Handbook of Chemistry*, McGraw-Hill Education, **2017**.

[11] D. R. Lide, *CRC Handbook of Chemistry and Physics*, CRC Press, **2004**.

[12] M. J. Kadhim, M. I. Gamaj, *J. Chem. Rev.* **2020**, *2*, 182–188.

[13] N. Q. Khuyen, Z. Zondaka, M. Harjo, J. Torop, T. Tamm, R. Kiefer, *Polymers* **2019**, *11*, 849.

[14] K. Xu, S. P. Ding, T. R. Jow, *Nonaqueous Electrolyte Development for Electrochemical Capacitors* **1999**.

[15] N. G. Tsierkezos, A. I. Philippopoulos, *Fluid Phase Equilib.* **2009**, *277*, 20–28.

[16] S. A. M. Refaey, G. Schwitzgebel, *Des. Monomers Polym.* **2000**, *3*, 389–398.

[17] H. Wang, X. Zhang, Q. Wu, F. Cao, D. Yang, Y. Shang, Z. Ning, W. Zhang, W. Zheng, Y. Yan, S. V. Kershaw, L. Zhang, A. L. Rogach, X. Yang, *Nat. Commun.* **2019**, *10*, 1–10.

[18] J. B. Milne, *Can. J. Chem.* **2011**, *58*, 283–286.

[19] P. K. Muhuri, D. K. Hazra, *ZNA* **1993**, *48*, 523–528.

[20] T. Uchino, G. N. Ayre, D. C. Smith, J. L. Hutchison, C. H. de Groot, P. Ashburn, *Nanomater.* **2021**, *11*, 2481.

[21] V. Sivasubramaniyam, S. Ramasamy, M. Venkatraman, G. Gatto, A. Kumar, *Energies* **2023**, *16*, 3665.

[22] N. Oppel, P. Röse, S. Heuser, M. Prokein, U. P. Apfel, U. Krewer, *Electrochim. Acta* **2024**, *490*, 144270.

[23] A. S. Kumar, M. Pupo, K. V Petrov, M. Ramdin, J. R. Van Ommen, W. De Jong, R. Kortlever, *J. Phys. Chem. C* **2023**, *127*, 12857–12866.

[24] T. Mairegger, H. Li, C. Grießer, D. Winkler, J. Filser, N. G. Hörmann, K. Reuter, J. Kunze-Liebhäuser, *ACS Catal.* **2023**, *13*, 5780–5786.
